# Early skin contact combined with mother’s breastfeeding to shorten the process of premature infants ≤ 30 weeks of gestation to achieve full oral feeding: the study protocol of a randomized controlled trial

**DOI:** 10.1186/s13063-021-05605-x

**Published:** 2021-09-17

**Authors:** Liling Li, Li Wang, Conway Niu, Chan Liu, Tianchan Lv, Futing Ji, Ling Yu, Weili Yan, Ya Lan Dou, Yin Wang, Yun Cao, Guoying Huang, Xiaojing Hu

**Affiliations:** 1 Children’s Hospital of Fudan University, 399 Wan Yuan Road, Shanghai, 201102 China; 2 King Edward Memorial Hospital, Western Australia Subiaco, Australia

**Keywords:** Skin contact, Breastfeeding, Preterm infants, Full oral feeding, Randomized controlled trial

## Abstract

**Background:**

Most hospitalized preterm infants experience difficulties in transitioning from tube feeding to full oral feeding. Interventions to promote full oral feeding in preterm infants in the neonatal intensive care unit (NICU) are limited to pacifier use or bottle-feeding exercises. Skin contact has been shown to be beneficial to start and maintain lactation and provide preterm infants with the opportunity to suck on the mother’s breast, which may promote further development of the preterm infant’s suckling patterns. The objective of this study is to compare and evaluate the effects of skin contact combined with breastfeeding (suck on the mother’s empty breast) as compared to the routine pacifier suckling training model in achieving full oral feeding for infants whose gestational age are ≤ 30 weeks.

**Methods:**

This is a single-center, randomized controlled clinical trial conducted in the NICU and designed according to the SPIRIT Statement. The subjects included in the study are premature infants born between April 2020 and July 2021 with a gestational age of ≤30 weeks, birth weight of <1500 g, admission age of <72 h, and absence of congenital malformations. Those with oxygenation indices of >40 and those born to mothers with poor verbal communication skills will be excluded. A sample of 148 infants is needed. The infants will be randomized to the intervention (skin contact combined with mother’s breastfeeding model) or control group (routine pacifier sucking training model). The primary outcome is the time required to achieve full oral feeding. The secondary outcomes are the breastfeeding abilities of preterm infants as assessed by the Preterm Infant Breastfeeding Behavior Scale (PIBBS), breastfeeding rates at 3 and 6 months corrected gestational age, complication rates, duration of oxygen requirement, days of hospital stay, and satisfaction of parents.

**Discussion:**

This paper describes the first single-center, open-label, randomized clinical trial on this topic and will provide crucial information to support the implementation of skin contact combined with the breastfeeding model in the NICU setting.

**Trial registration:**

ClinicalTrials.gov NCT 04283682. Registered on 8 February 2020.

**Supplementary Information:**

The online version contains supplementary material available at 10.1186/s13063-021-05605-x.

## Background

Oral feeding is a complex sensory motor process which is affected by many factors. The brain of the preterm infant is not fully developed and thus neural development is incomplete. Oral feeding is difficult due to decreased abilities in sucking and swallowing, discordant breathing, and immature behavior state organizational capabilities [1, 2]. Studies report that 80% of preterm infants experience oral feeding difficulties with the degree of feeding difficulty related to gestational age [3, 4]. Although sucking and swallowing appears at 28 weeks of gestation, full coordination of sucking, swallowing, and breathing should be achieved at between 32 and 34 weeks’ gestation, which means that preterm infants less than 32 weeks of gestation are unable to feed orally by breast or bottle [4–9].

During neonatal intensive care unit (NICU) admission, preterm infants often require nasogastric or orogastric tube feeding before progressing to oral feeds once they are mature enough to do so [10]. Preterm infants’ readiness for full oral feeding is related to gestational age. According to research data on preterm infants at different gestational ages, full oral feeding was achieved at 10–36 days of life [11–14]. Establishment of full oral feeding in preterm infants is an important factor affecting the length of hospital stay and is also one of the criteria considered for discharge from the NICU [15–18].

Studies have shown that preterm infants can achieve full oral feeding before 34 weeks’ gestation through external stimulation (e.g., pacifier, lullabies, music, the smell of breast milk, kangaroo care) [19]. Oral feeding training can improve the experience and maturity of sucking [4, 20–23]. Preterm infants show obvious signs of rooting, grasping, and repeated short sucking at 29 weeks’ gestation and occasionally long sucking and repeated swallowing at 31 weeks’ gestation. Regular training in non-nutritive sucking can allow the preterm infant to gain experience in feeding, promote the formation of mature sucking, and contribute to the development of feeding skills [2, 4]. Nyquist et al. found that babies can root, latch, and effectively contain the areola at 28 weeks of gestation when they come into contact with their mother’s breasts [24]. There was no correlation between the rooting behavior, sucking, and gestational age. After 30 weeks’ gestation, preterm infants can exhibit nutritive sucking. Repeated bursts of more than 10 sucks and maximum bursts of more than 30 sucks are observed at 32 weeks [24].

Non-nutritive sucking by sucking the mother’s “empty” breast is more conducive to promoting the development and maturity of sucking than using a pacifier. This sucking is completed at the source of nutrition without affecting nutrient intake. When sucking the “empty” breast, the baby showed that the nipple was tightly locked and fixed on the nipple. Time spent in active sucking, sucking burst, and swallowing were all longer and feeding behavior was more mature [25]. Skin-to-skin contact (SSC) is a maternal and infant practice recommended by the World Health Organization (WHO). It has been proven that SSC plays an important role in both infants and mothers [26]. The sleep or wakefulness behavior of infants receiving SSC is more organized, and they have more time in quiet sleep and in the alert awake state [27, 28]. SSC contributes to the development of the infant’s nervous system [28–30], and it has been reported that infants receiving SSC have shorter hospital stays and lower infection severity [31, 32]. Infants who received SSC had higher intelligence and better psychomotor development at 12 months corrected gestational age [30]. From the breastfeeding perspective, SSC can improve the production of breast milk, help establish breastfeeding, prolong the duration of breastfeeding, enhance the willingness to breastfeed [33–35], and help to reduce stress in new mothers [35].

In the process, the mother expresses breast milk prior to tube feeding and tube feeding is combined with skin-to-skin contact. The mother manually drips the breast milk onto the baby’s lips, and the baby tastes and becomes familiar with breast milk while being tube-fed. Non-nutritive sucking on the breast is conducive to the transition from tube feeding to breastfeeding, which is in turn conducive to breastfeeding [36]. As the infant matures, the infant will naturally approach the breast during SSC, which triggers instinctive breastfeeding behavior, promotes the coordination of sucking and swallowing, starts the first nutritive sucking earlier, and improves the duration of sucking, so as to satisfy the infant’s instinctive desire to suck.

At present, many NICUs promote oral feeding of preterm infants mainly by non-nutritive sucking and tube feeding, and the outcome indicator is predominantly successful establishment of bottle feeding. Studies on skin contact in stable preterm infants mainly focus on temperature management, weight gain, breast milk secretion, and parental satisfaction. Family-centered care focuses more on the interaction between parents and preterm infants during hospitalization and while in transitional care before discharge. In the early stages of establishing feeds, the lack of participation of lactating mothers and the loss of the most natural active feeding support led to the prolonging of the feeding process and of hospital stay.

Therefore, the primary objective of this study is to explore whether empty breast sucking combined with skin contact can shorten the process of full oral feeding of preterm infants with gestational age ≤ 30 weeks.

## Methods/design

### Study setting and design

**Fig. 1:**
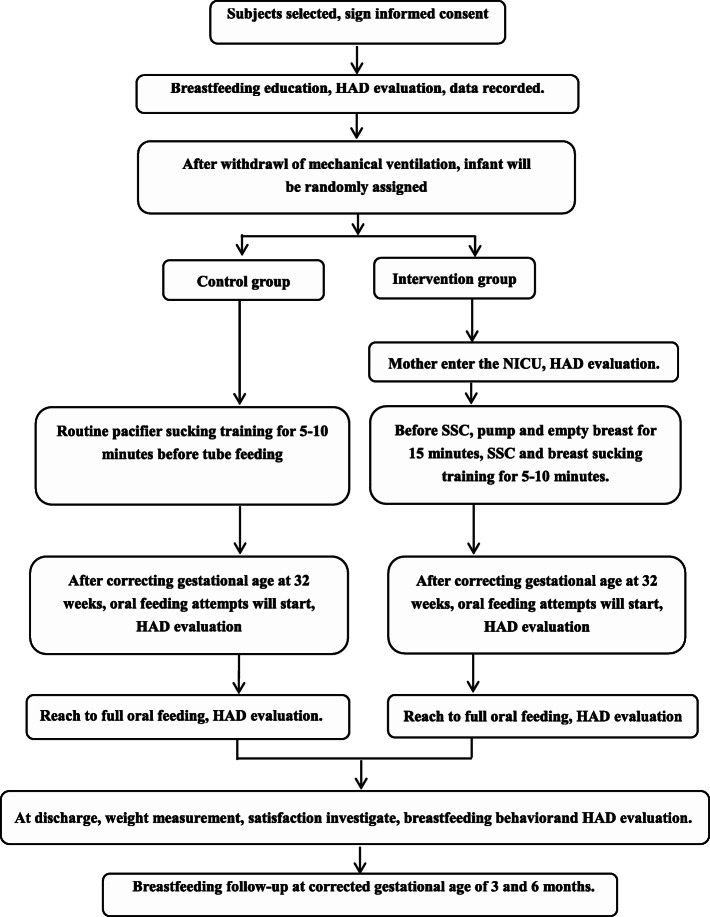
The procedure of the trial

We will conduct a randomized, open-label clinical trial in the NICU at the Children’s Hospital of Fudan University (CHFU) in Shanghai, China. CHFU has a capacity of 1000 beds with a large neonatal center consisting of 80 NICU beds. Patients requiring admission to the NICU are those who are especially critical, approximately 2200 annually. These patients are transported from all regions of the country, with a higher proportion from the Yangtze River Delta region. More than half of these patients are preterm with approximately 500 very low birth weight infants admitted to the CHFU annually. This protocol refers to the SPIRIT 2013 Statement (Standard Protocol Items: Recommendations for Interventional Trials). A SPIRIT checklist is seen in Additional file 1. The procedures of the trial are presented in Fig. 1.

### Study subjects and recruitment

Preterm infants will be treated in the NICU at the CHFU meeting the following inclusion criteria:

- Gestational age ≤ 30 weeks
- Birth weight < 1500g
- Admission age < 72h
- No congenital malformations

Exclusion criteria:

- Oxygenation index (OI = 100 × mean airway pressure (MAP) × FiO_2_/PaO_2_) > 40
- Mother unable to communicate adequately
- Twins or multiple births

Dropout criteria:

- Death or withdrawing treatment during hospitalizing
- Mothers are unable to provide empty breast sucking or skin contact due to illness or other reasons
- Premature infants cannot receive oral feeding due to disease

### Management before procedure

Before the start of the study, NICU nurses will undergo training in the theory of and technical training in kangaroo care and breastfeeding in preterm infants. On the day of admission, eligibility will be determined according to the inclusion and exclusion criteria. The breastfeeding education nurse conducts breastfeeding education and sends the Hospital Anxiety and Depression Scale anxiety subscale (HAD) [37] questionnaire to the mother for completion within 24 h by WeChat, China’s predominant social media platform. The questionnaire will be withdrawn and made unavailable after 24 h. After admission, respiratory support, enteral and parenteral nutrition, lactation volume, and weight will be recorded daily until the infant is discharged home.

### Consent

Explanations are made with consideration of potential emergency situations, and explanations of the purpose, methods, and possible expectations of the study are made to the mothers who meet the inclusion criteria. Informed consent will be obtained from parents.

### Interventions

#### The control group

After withdrawing mechanical ventilation, before each feed, a pacifier is used for 5–10 min of non-nutritive sucking. Feeds are given with a special bottle designed for preterm infants. Body weight is measured before the first oral feeding is given. When oral feeding is commenced, the infant’s mother will be informed by telephone that this has started. At this point, the HAD questionnaire is sent to the mother for a second time for completion within 24 h. According to the oral feeding schedule, the frequency of oral feeds is gradually increased. Full oral feeding is considered established when the daily amount of oral feeding milk reaches 75% of the required volume, no gavage feeding is needed for 48 h, and the daily body weight increases by 10–15g/kg/day. At this time, the infant is weighed, the mother is informed by phone, and the HAD questionnaire is sent to the mother for the third time for completion within 24 h. The weight will be measured again on the day of discharge, and parental satisfaction will be assessed by EMPATHIC-N [38]. On discharge, the HAD questionnaire was sent to the mother for the fourth time for completion within 24 h. And the PIBBS scale will be used to evaluate the breastfeeding behavior of preterm infants on discharge day. The research team members will organize and enter data on the infant’s clinical course in the hospital, and a second-team member will be responsible for verifying the data collection and entry. After discharge, the rate and mode of breastfeeding will be followed up by breastfeeding education nurses at 3 and 6 months corrected gestational age.

#### The intervention group

After the withdrawal of mechanical ventilation, the infant will be assessed for 12 to 24 h and participants in the intervention group would receive a combination of skin-to-skin contact and breastfeeding. When the infant’s condition is stable, the mother will be invited to enter the NICU, and the HAD questionnaire will be sent to the mother for the second time for completion within 24 h. Before SSC, the mother pumps and expresses breast milk for about 15 min. Nurses assist the SSC process and carry out empty breast sucking (suck on empty breast) training for 5 to 10 min. In case of non-skin contact, a pacifier shall be used for sucking before tube feeding. SSC will be carried out at least once a day for 1 h, and monitoring should be done at the same time. The infant will be fed by a nasogastric tube. At 32 weeks’ gestation, attempts will be made to feed the infant by the bottle, using bottles specially designed for preterm infants. Before feeding, non-nutritive sucking will be conducted with a pacifier for 5 to 10 min. The number of oral feeding attempts will be gradually increased according to the oral feeding schedule. Achievement of full oral feeding is achieved when the daily amount of oral feeding milk reaches 75% of the required volume, no gavage feeding is needed for 48 h, and the daily body weight increases by 10–15g/kg/day. At this point, the infant is weighed, SSC is ceased, and the HAD questionnaire is sent to the mother for the third time for completion within 24 h. The weight will be measured on this day. On the day of discharge, the infant is weighed, and parental satisfaction will be assessed by EMPATHIC-N [38]. And the PIBBS scale will be used to evaluate the breastfeeding behavior of preterm infants on discharge day. The HAD questionnaire is sent to the mother for the fourth time at this time for completion within 24 h. Research team members will complete the organization and entry of the infant’s clinical course in the hospital, and a second-team member will be responsible for verifying the collection and entry of the information. After discharge, the rate and mode of breastfeeding will be followed up by breastfeeding education nurses at 3 and 6 months corrected gestational age.

### The nutritional protocol in the two groups

Direct breastfeeding can be carried out after discharge without adding a fortifier. For both groups, lactation consultants will deliver information on the benefits of breastfeeding to mothers at admission, provide breastfeeding practice before discharge, provide neonatal follow-up after discharge, and provide breastfeeding guidance at the outpatient clinic. Breast milk fortification begins when the amount of breast milk reaches 50~80 ml/(kg day). A breast milk fortifier needs to be used immediately after configuration.

### Outcomes and measurements

#### Primary outcome

The time from the first oral feeding to full oral feeding (days) [39]: When the daily amount of orally fed milk reaches 75% of the required volume, no gavage feeding is needed for 48 h, and the daily body weight increases by 10–15g/kg/day, the infant is defined as having reached full oral feeding.

#### Secondary outcomes

- The proportion of breastfeeding at discharge: Based on the record on CRF, we will calculate the number of infants who are exclusively breastfeeding without formula top-ups.
- The exclusive breastfeeding rate at 3 and 6 months after discharge: Breastfeeding status will continue for 3 or 6 months or until mixed feeding is established [40].
- Breastfeeding ability of preterm infants: The assessment of rooting, areolar grasp, duration of latching on, sucking, sucking burst, and swallowing [41]. It will be evaluated on discharge day. The Preterm Infant Breastfeeding Behavior Scale (PIBBS) will be used and the scale was developed by professionals and mothers of preterm infants [41].
- Length of hospital stays: Based on the record on CRF, we will calculate the duration of each subject from NICU admission to discharge.
- The number of complications: Based on the record on CRF, we will calculate the number of infants who have complications as follows:
  - ♦ Intraventricular hemorrhage (IVH) diagnosed by cranial ultrasound [42]
  - ♦ Retinopathy of prematurity (ROP) diagnosed by funduscopic examination [43]
  - ♦ Bronchopulmonary dysplasia (BPD) defined by an oxygen requirement that persists beyond 36 weeks’ gestational age [44]
  - ♦ Necrotizing enterocolitis (NEC) diagnosed by identifying pneumatosis, intrahepatic portal venous gas, or free air by radiograph [45]
  - ♦ Sepsis diagnosed by blood culture [46]
  - ♦ Patent ductus arteriosus (PDA) diagnosed by cardiac ultrasound [47]
- Days requiring supplemental oxygen: Based on the record on CRF, we will calculate the days of oxygen therapy in any form for each subject.
- The degree of anxiety and depression in parents: In the Hospital Anxiety and Depression Scale anxiety subscale, A represents anxiety, while D represents depression, with each item divided into four grades. The total scores of anxiety and depression are obtained by superposition. A total score of 0–7 is normal, 8–10 indicates mild depression or anxiety, 11–14 indicates moderate depression or anxiety, and 15–21 indicates severe depression or anxiety [37].

### Sample size

Sample size calculation is performed based on the primary outcome that is the time to achieve full oral feeding. The average days to achieve full oral feeding was 14 days with a standard deviation of 7 days in the control group according to the previous study. The time for the intervention group to achieve full oral feeding was 4 days shorter than that of the control group [48]. The sample size of 66 in each group could achieve 90% power to detect a difference of 4.0 between the groups with estimated group standard deviations of 7.0 and with a significance level (alpha) of 0.05. At least 74 subjects will be recruited in each group considering a 10% drop rate. Sample size calculation was performed by Power Analysis & Sample Size ® software version 16.0 (NCSS, LLC. USA).

### Randomization

The subjects will be randomly allocated to the intervention group or the control group by block randomization method with a block size of 4 and allocation of 1:1. The randomization will be done by SAS version 9.4 (SAS Institute, Inc). A sequence of subjects’ IDs and intervention allocations will be generated, which by creating a random variable and ordering on that variable. The allocation sequence will be concealed by the statistician and placed in sequentially numbered, opaque, sealed, and stapled envelopes. After signing the informed consent from parents, the study investigators will open the envelopes in consecutive orders and randomly assign the subjects to the intervention group or the control group according to the allocation scheme in the envelope. The staff who analyze the outcome is unaware of the allocation of subjects.

### Data collection, management, analysis, auditing

Source of data acquisition:

- The infants and the mother’s medical history will be obtained from the medical records
- Infants’ vital signs from the nursing information system
- Feeding information and physical situation will be recorded in nursing documents
- Skin contact and PIBBS will be assessed using real-time recordings of group members
- Information on correcting breastfeeding for gestational age of 3–6 months will be obtained from the mother’s telephone consultation

An electronic information collection form will be designed, and the team members will be responsible for recording all data on the paper of the day in the first instance.

The clinical research assistant is in charge of entering all the data in an electronic case report form (CRF). Another member of the research team verifies the newly entered data on paper every week. Another clinical research assistant checks the data of each case individually. The data can only be used after the completion of the study, with the data remaining anonymous prior to this. A clinical research assistant mandated by the sponsor will regularly supervise the study process every month. The elements to be audited during these visits will be defined prior to the start of the study. The results of each surveillance process need to be documented in writing, and epidemiological and statistical experts will be invited to work together to improve issues on a monthly basis. The database will be built using Access 2013 (Microsoft office Professional 2016); double data entry and check will be performed by the trained data entry staff.

### Statistical analyses

All analyses will be performed using SPSS 22.0 (IBM SPSS Statistics, IBM Corporation, Chicago, IL). The two-tailed significance level will be set to *P* < 0.05. An intention-to-treat (ITT) analysis will be used for the outcomes. For primary outcome analyses, the generalized linear model (GLM) will be used to test the difference of the time to achieve full oral feeding (days) between the groups, and the estimated means and two-sided 95% confidence intervals (CI) will be derived. The clinical factors including gestational age, sex, birth weight, admission age, oxygenation indices (OI), etc. will be adjusted in the covariate-adjusted analysis of the primary outcome by the GLM model. If any subjects dropped off from the primary endpoint, modified ITT analysis will be performed by using the available subjects.

All secondary outcomes will be analyzed by GLM. Binary secondary outcomes will be summarized as absolute numbers and percentages, and the difference and its 95% CIs between the two groups will be estimated. The continuous outcome will be presented as means and standard deviations (SD) and be analyzed with the same strategy and method with that is used for the primary outcome.

### Harms

Each infant included in the study will have a monitor for continuous monitoring, and special nursing staff will take care of the infants at the bedside. Once any unexpected events occur, such as desaturation, bradycardia, vomiting, thermal instability, or any event that leads to the interruption of stimulation, the medical team of Neonatology will record the occurrence type, time, and treatment measures in detail and carry out emergency measures to ensure the safety of the infants.

### Auditing

The sponsor selects personnel independent of the trial as auditors to conduct a systematic and independent examination of the trial-related activities and documents regularly.

The contents to be confirmed in the audit process include (1) rights and safety of subjects; (2) data are accurate, complete, and verifiable from source documents and accurately analyzed and reported; and (3) conduct of the trial is in compliance with the protocol, standard operating procedure, and applicable regulatory requirements.

After the auditing, all inspection findings and deficiencies need to be documented and saved. The sponsor or investigator needs to submit the response to the deficiencies and the planned improvement action to the inspector.

### Research ethics approval

The study (2019) 295 was approved by the Ethics Committee of CHFU on Dec 25, 2019. If the research protocol needs to be modified, team members and epidemiology and statistics experts will be consulted, and the protocol will be re-submitted to the Ethics Committee. Team members will be retrained if necessary.

### Protocol amendments

Protocol amendments that must be reported include the following:

- Any increase in intervention duration beyond that described in the current protocol, or any significant increase in the planned number of subjects enrolled.
- Any significant change in the design of a protocol (such as the addition or elimination of a control group).
- Addition of a new test or procedure intended to improve monitoring for, or reduce the risk of, a side effect or adverse event; or elimination of a test intended to monitor safety.

The amended study protocol with a description of the changes should be recorded as the new version in detail, then submitted to the ClinicalTrials.gov and the local Ethics Committee for approval.

### Ancillary and post-trial care

Participants enrolled into the study will receive the following additional health care during the trial: provide breast milk collection storage and transportation training, daily telephone consultation and communication, and breastfeeding or hand hygiene training manual. After the completion of the trial, the investigator will continue to provide post-trial access and ancillary care to the participants, including giving instructions on breastfeeding before discharge, both groups will have access to detailed home care education on day of discharge, and providing an outpatient care for premature infants. If participants experience trial-related harms, the investigator will provide them with compensation or additional care.

## Discussion

Oral feeding is the optimal means of feeding for preterm infants, and the establishment of full oral feeding is the ultimate goal for these infants. The delay of full oral feeding is one of the main reasons for delayed discharge [49, 50]. However, the early realization of full oral feeding of preterm infants is challenging. Preterm infants need to coordinate sucking, swallowing, and breathing, as well as maintain vigilant awake behavior and cardiopulmonary stability to achieve full oral feeding [7]. Based on experience, gestational age is the standard to determine the commencement of oral feeding [51] and it is safer to introduce oral feeding when the coordination of sucking, swallowing, and breathing occurs at 32–34 weeks of gestation. Nyqvist et al. carried out a study on breastfeeding of very low birth weight preterm infants, which showed that the preterm infants at 29 weeks’ gestation had obvious rooting and occasional sucking performance. The nipples and areola could be grasped at 30 weeks’ gestation, and at 31 weeks’ gestation, the breast latching of more than 15-min duration, with repeated short sucking and swallowing sounds observed [52]. Pickler et al. found that the maturity of oral feeding and feeding experience are important factors which could affect the success of full oral feeding. The time at which oral feeding first occurs can predict the discharge time of infants [2]. Regardless of how poor the infant’s clinical condition is, active oral feeding exercise will help the transition to full oral feeding. Therefore, oral feeding does not have to wait until 32–34 weeks. Achieving earlier full oral feeding in preterm infants is a priority for neonatal medical staff.

If preterm infants are not given opportunities to use their sucking reflex, this reflex will be at risk of being lost [53]. Non-nutritive sucking has been proven to promote the coordination of sucking and swallowing [54], accelerate the maturation of the sucking reflex [55], improve the start and duration of nutritive sucking [56], increase body weight [57], and help preterm infants transition from nasogastric feeding to oral feeding earlier. The best pacifier is a mother’s nipple. Early postnatal skin-to-skin contact (SSC) is an internationally recommended practice [58]. It is very important for infants and mothers to maintain cardiopulmonary stability and to commence early breast latching to promote breastfeeding. The first stage of preterm oral movement behavior is licking and tasting. When the preterm infant comes into contact with the mother’s breast, the posture the mother uses to hold the infant and adjustment of this posture with respect to the breast can make the preterm infant latch on the breast more effectively and fix on it. The suck reflex in preterm infants is triggered by approaching the mother, being held by the mother, and latching on the mother’s nipple to exercise their sucking ability. Preterm infants suck their mother’s empty breasts through early contact with the mother’s skin, which not only satisfies the infant’s desire to suck and contributes to the development of a more mature sucking pattern, but also improves breast milk secretion and the rate of breastfeeding [59].

This study will provide data on the feasibility for preterm infants to undergo skin contact and mother’s parent feeding immediately after weaning of mechanical ventilation in the NICU and evaluate the effect of early SSC combined with suckling on an empty breast on shortening the time for preterm infants ≤ 30 weeks’ gestational age to achieve full oral feeding. It will provide evidence to support the clinical practice of SSC combined with suckling on an empty breast to promote full oral feeding.

## Trial status

The protocol version number is ClinicalTrials.gov, and the identifier is NCT04283682 Children’s Hospital of Fudan University. Protocol record is EKYY-2019-295. The registration date was on February 8, 2020. The date recruitment began on April 8, 2020. And the approximate date when recruitment will be completed is July 1, 2021.

## Supplementary Information

**Additional file 1.** SPIRIT 2013 Checklist: Recommended items to address in a clinical trial protocol and related documents.

## Data Availability

The data recorded during this research are subject to computer processing by the clinical research center, Children’s Hospital of Fudan University, in accordance with Law No. 78–17 of 6 January 1978 on computers, files, and freedoms amended by Law 2004–801 of August 6, 2004.

## Abbreviations

NICU: Neonatal intensive care unit
PIBBS: Preterm Infant Breastfeeding Behavior Scale
SSC: Skin-to-skin care
CHFU: Children’s Hospital of Fudan University
OI: Oxygenation index
HAD: Hospital Anxiety and Depression Scale anxiety subscale
IVH: Intraventricular hemorrhage
ROP: Retinopathy of prematurity
BPD: Bronchopulmonary dysplasia
NEC: Necrotizing enterocolitis
GA: Gestational age
